# Association Between Socioeconomic Factors and the COVID-19 Outbreak in the 39 Well-Developed Cities of China

**DOI:** 10.3389/fpubh.2020.546637

**Published:** 2020-10-30

**Authors:** Yiting Lin, Ping Zhong, Ting Chen

**Affiliations:** ^1^Department of Respiratory and Critical Care Medicine, Xiamen Haicang Hospital, Xiamen, China; ^2^BE and Phase I Clinical Trial Center, The First Affiliated Hospital of Xiamen University, Xiamen, China; ^3^Department of Medical Examination and Blood Donation, Xiamen Blood Center, Xiamen, China

**Keywords:** SARS-CoV-2, public health emergency of international concern, emerging infections diseases, urbanization, gross domestic product, population density, internal migration

## Abstract

**Background:** Socioeconomic factors play an indispensable role in the spread of emerging infectious diseases. Few studies have investigated the role of socioeconomic factors in the spread of COVID-19.

**Methods:** The number of COVID-19 cases in the 39 well-developed cities of China was aggregated by searching the publicly available sources. Socioeconomic indicators (e.g., population, population density, gross domestic product, rural-to-urban migrants, urbanization rate, per-person disposable income, and level of health care) in these cities were also aggregated from the Bureau of Statistics. The data referring to travelers from Wuhan were collected from the Baidu Migration database. A multiple stepwise linear regression model was performed to identify the independent risk factors of the number of cases.

**Results:** As of Mar 19, 2020, a total of 5,939 cases were reported in the 39 well-developed cities with almost half of total cases in China outside of Hubei. The number of cases ranged 20–576, and the median number of cases was 93 (IQR 54–180) in these cities. Nine socioeconomic variables including the number of travelers from Wuhan, population, native population, gross domestic product, Per-person GDP, the number of hospitals, the number of rural-to-urban migrants, traffic capacity, and person-disposable income were recognized as potential contributors of the number of cases. Results of multiple linear regression showed a statistically significant association between the number of cases and the number of travelers from Wuhan (*t* = 6.746, *P* = 0.000) and the number of rural-to-urban migrants (*t* = 3.776, *P* = 0.001) in these cities. However, other seven potential contributors were not associated with the number of cases. Moreover, a well-fitted multiple regression model was built in this study, and a regression equation was as follows: Y = 0.007X_t_ + 0.200X_m_ (adjusted *R*^2^ = 0.833).

**Conclusions:** Travelers from Wuhan and rural-to-urban migrants were independently associated with the COVID-19 outbreak in the 39 well-developed cities of China. These findings suggested that travelers from an epicenter and rural-to-urban migrants should be paid more attention in the early stage of the COVID-19 outbreak in the well-developed cities.

## Introduction

The ongoing coronavirus disease 2019 (COVID-19) outbreak is a challenge for global health governance. As of Sep 20, 2020, tens of millions of confirmed cases have been reported globally, with total deaths exceeding 950,000 (1). The World Health Organization increased the assessment of the risk of spread and risk of COVID-19 to very high at the global level, and characterized it as a pandemic (1). Thus, the epidemic investigation on the COVID-19 outbreak is meaningful and urgent due to its high rate of spread and significant number of fatalities.

In China, Wuhan was the epicenter where the COVID-19 outbreak happened. In the early stage of an outbreak, most of the reported cases in other cities were geographically associated with Wuhan. The local government implemented a lockdown of Wuhan on Jan 23, 2020 owing to the outbreak of COVID-19. In our previous research, we found that the travelers departing from Wuhan before Jan 24, 2020 were significantly associated with the number of the confirmed cases in other cities of China (2). However, compared with other popular destination cities, the disproportionately more cases were also observed in several well-developed cities, such as Beijing, Shanghai, Guangzhou, Shenzhen, Tianjin, and Hangzhou (2). Moreover, only about a third of confirmed cases in Tianjin were geographically associated with Wuhan, indicating that community transmission of COVID-19 largely occurred in the city (3). Furthermore, a large number of COVID-19 cases were reported in the metropolises of China (e.g., Beijing, Shanghai), and the number still increased every day when no new cases were identified in most regions of China. All these phenomena indicated that the travelers form Wuhan may not be the sole factor, and there were some other unknown risk factors which induced the COVID-19 outbreak in these well-developed cities. In general, demographic factors, population factors, economic factors, travel, and recreation are thought to be contributing factors to the occurrence of emerging infectious diseases (4). A huge population, high population density, increased population mobility, and rapid urbanization, which commonly exist in well-developed cities, are also the risk factors in the emergence and rapid spread of epidemic diseases (5, 6). Besides, it was suggested that urban income had a positive effect on the spread of severe acute respiratory syndrome (SARS), which is also caused by coronavirus (7). Hence, socioeconomic factors play an indispensable role in the spread of emerging infectious diseases. However, few studies have investigated the role of socioeconomic factors in the spread of COVID-19 so far. Therefore, the purpose of this study was to investigate the association between socioeconomic factors and the spread of COVID-19 in the 39 well-developed cities of China.

## Materials and Methods

### Socioeconomic Data Collection

Initially, 48 well-developed cities outside of Hubei Province were included according to the National Urban System Planning of China. Commonly, these cities are municipalities directly under the Central Government, capital cities, or regional centers. Certain potential socioeconomic factors affect infectious disease transmission in itinerant populations, and we selected these indicators based on the results of previous studies (4–7). Therefore, the socioeconomic variables analyzed in this study were as follows: population, native population, population density, regional gross domestic product (GDP), per-person GDP, the number of rural-to-urban migrants, the proportion of rural-to-urban migrants, urbanization rate, the proportion of tertiary industry, traffic capacity, per-person disposable income, the number of hospitals, and the number of doctors. Meanwhile, these indicators in China mainland outside Hubei Province were also aggregated. All these data were extracted from the National Bureau of Statistics of China (http://data.stats.gov.cn/) and the Statistical Communique on the 2018 national economic and social development issued officially by each municipal bureau of statistics in 2019.

### Migration Data Collection

Based on our previous study, the travelers departing from Wuhan before the Spring Festival were significantly associated with the number of the confirmed cases in other cities (2). Thus, the data of travelers from Wuhan for each city was also collected. The data referring to population mobility were collected from the Baidu Migration database (http://qianxi.baidu.com/), which is a non-profit project aiming to provide data on population mobility during the Chinese Spring Festival rush. This database is obtained from the location-based services database of Baidu Company, which helps it become the most accurate migration database in China at present. This database is able to provide the overall proportion of travelers departing from one city to the top 100 cities during a certain period. According to the instructions of the database, the proportion of travelers departing from Wuhan to one city is calculated by n_1_/n_2_, where n_1_ is defined as the number of travelers departing from Wuhan to one selected city during a certain period and n_2_ is the total number of travelers departing from Wuhan during a certain period. For instance, the proportion of travelers departing from Wuhan to Beijing was 1% if the number of travelers departing from Wuhan to Beijing was 50,000 during a certain period and the total number of travelers departing from Wuhan was 5 million during the same period. The annual Spring Festival travel rush started on Jan 10, 2020. Meanwhile, Wuhan was completely locked down on Jan 24, 2020 (the day before the Spring Festival). Thus, between Jan 10, 2020 and Jan 24, 2020, the proportions of travelers departing from Wuhan to the top 100 cities were extracted. However, only 39 well-developed cities were in the top 100 destination cities. Thus, the proportions of travelers departing from Wuhan to the 39 well-developed cities were extracted. In addition, it was announced by the mayor of Wuhan city that about 5 million people left the city during the Chinese Spring Festival rush (from Jan 10, 2020 through Jan 24, 2020). Therefore, the estimated number of travelers from Wuhan in a destination city was calculated by 5 million multiplied by the proportion of travelers departing from Wuhan to the selected city.

### Case Data Collection

All confirmed cases of COVID-19 in the 39 well-developed cities and in China outside of Hubei Province were aggregated from official announcements by searching the publicly available sources (the situation report of COVID-19 announced by each Municipal Health Commission and National Health Commission of China). The deadline was at 11:59 P.M. on Mar 19, 2020 (China standard time, CST). According to the official announcements, all cases were applied with the same diagnostic criteria based on the recommendation by the National Health Commission of China (http://www.nhc.gov.cn/). The confirmed cases conformed to the Case Definitions for Surveillance of COVID-19 as follows: (1) a suspect case was diagnosed clinically; (2) a suspect case of COVID-19 that is positive for SARS-Cov-2 by RT-PCR and sequencing at the City and Provincial Centers for Disease Control and Prevention. No ethical approval was needed for this study.

### Statistical Analysis

Continuous variables were expressed as median and interquartile range (IQR). We used an analysis of correlation to evaluate the associations between the number of cases and the number of travelers from Wuhan and other socioeconomic factors in the 39 well-developed cities. If the data were not normally distributed, a Spearman correlation analysis was applied. In addition, the scatter plots and the regression curves were drawn to test the linearity assumption. Furthermore, the factors producing correlations with *P* < 0.15 were then used in a multiple stepwise linear regression model (through the origin) to identify the socioeconomic factors most strongly associated with the number of COVID-19 cases. The data were analyzed by SPSS statistic 22.0 (SPSS Inc., Chicago, USA). All statistical significance was defined as *P* < 0.05.

## Results

### Basic Characteristics of Socioeconomic Indicators and COVID-19 Cases in the 39 Well-Developed Cities

**Figure 1:**
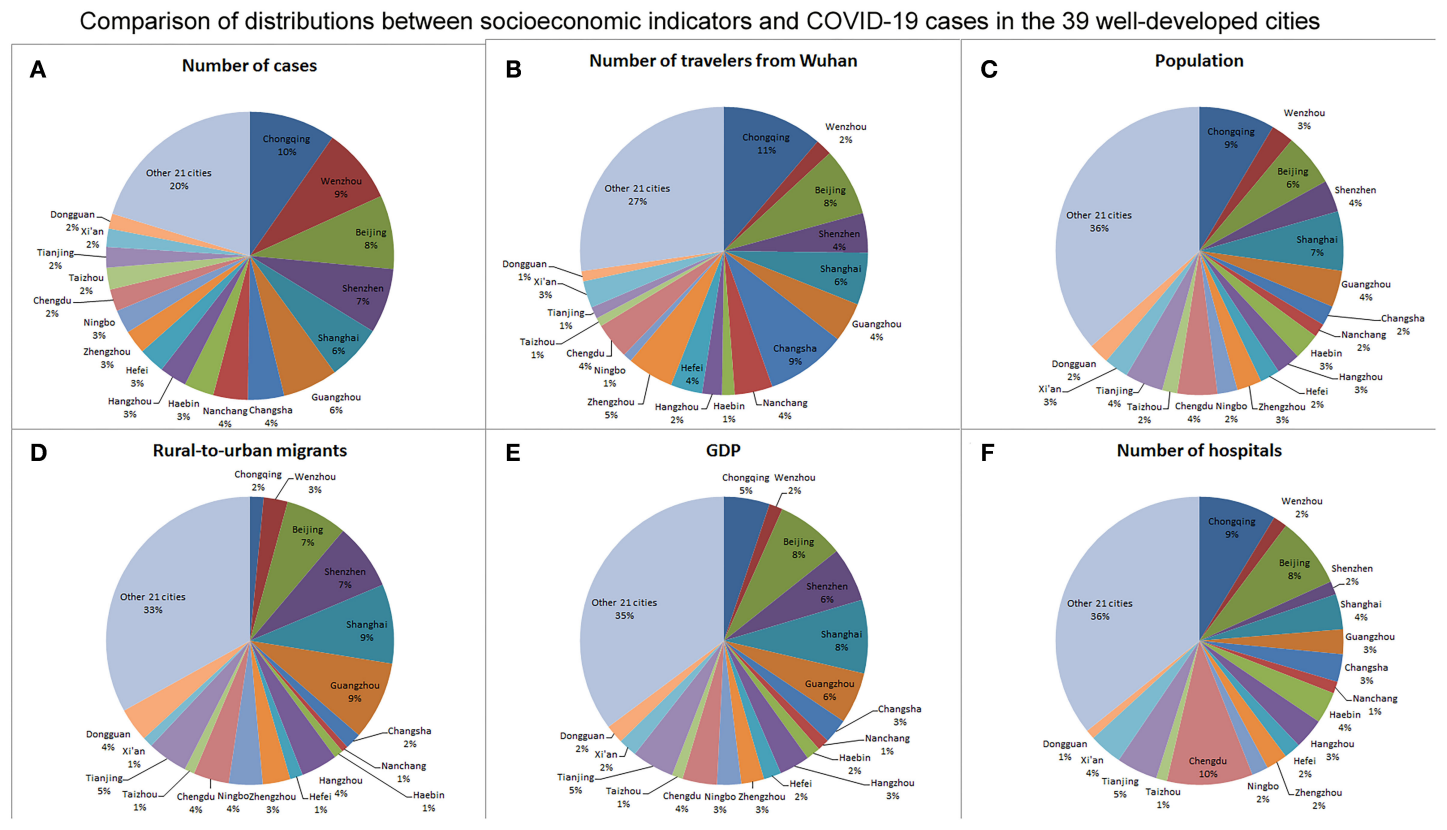
**(A)** Comparison of basic characteristics of socioeconomic indicators and COVID-19 cases. The distribution of COVID-19 cases roughly companied with the distributions of travelers from Wuhan **(B)**, population **(C)**, GDP **(E)**, rural-to-urban migrants **(D)**, and hospitals **(F)** in the 39 well-developed cities. The proportions shown in the figure represent the proportions of each city in the 39 well-developed cities.

Table 1 shows the basic characteristics of socioeconomic indicators and COVID-19 cases in the 39 well-developed cities. There were four municipalities directly under the Central Government (Beijing, Shanghai, Tianjin, and Chongqing), 20 capital cities (e.g., Guangzhou, Hangzhou), and 15 regional centers (e.g., Shenzhen, Suzhou) in the 39 well-developed cities. The number of cases ranged 20–576, and the median number of cases was 93 (IQR 54–180). The median population was 8.2 million (IQR 5.73–10.57) while the median GDP was RMB 799.67 billion (IQR 481.18–1261.53). Per-person GDP and per-person disposable income were at a high level with a narrow interquartile range in these cities. In addition, the median number of rural-to-urban migrants was 1.97 million (IQR 1.21–3.87). Comparison of distributions between socioeconomic indicators and COVID-19 cases in the 39 well-developed cities is shown in Figure 1. The distribution of COVID-19 cases roughly companied with the distributions of travelers from Wuhan, population, GDP, rural-to-urban migrants, and hospitals in these cities.

**Table 1:** Basic characteristics of socioeconomic indicators and COVID-19 cases in the 39 well-developed cities.

**Variables, (median, interquartile range)**	**No. of cities**	**Variables, (median, interquartile range)**	**No. of cities**
Number of cases (*n*), 93 (54.63–180.76)			
<50	7	Proportion of tertiary industry (%),58.00 (50.88–61.37)	
50–99	14	<50	7
100–350	12	50–60	19
>350	6	>60	13
Number of travelers from Wuhan (*n*), 9000.00 (5694.00–19250.00)		Number of rural-to-urban migrants (10, 000), 196.67 (121.00–387.50)	
<8,000	17	<150	14
8,000–25,000	15	150–400	15
>25,000	7	>400	10
Population (10, 000), 820.00 (573.25–1057.25)		Proportion of migrants (%), 26.76 (17.37–44.24)	
<500	8	<20	12
500–1,000	20	20–40	16
>1,000	11	>40	11
Population density (person/km^2^), 830.97 (617.64–1057.25)		Urbanization rate (%),74.97 (70.07–82.98)	
<500	7	60–70	9
500–1,000	18	70–80	18
>1,000	14	>80	12
GDP (RMB 100 million), 7996.70 (4811.80–12615.30)		Native population (10, 000), 530.00 (386.50–759.50)	
<5000	11	<500	17
5,000–10,000	13	500–1,000	16
>10,000	15	>1,000	6
Per-person GDP (RMB 1,000), 97.32 (76.46–133.91)		Traffic capacity (million per year), 101.00 (58.56–199.68)	
<50	2	<50	6
50–100	19	50–150	19
>100	18	>150	14
Per-person disposable income (RMB 1,000), 42.99 (34.20–50.69)		Number of hospitals (*n*), 174 (115.25–308.75)	
<30	4	<100	7
30–50	23	100–300	22
>50	12	>300	10

### Comparisons of Socioeconomic Indicators and COVID-19 Cases Between the 39 Well-Developed Cities and China Outside of Hubei

**Figure 2:**
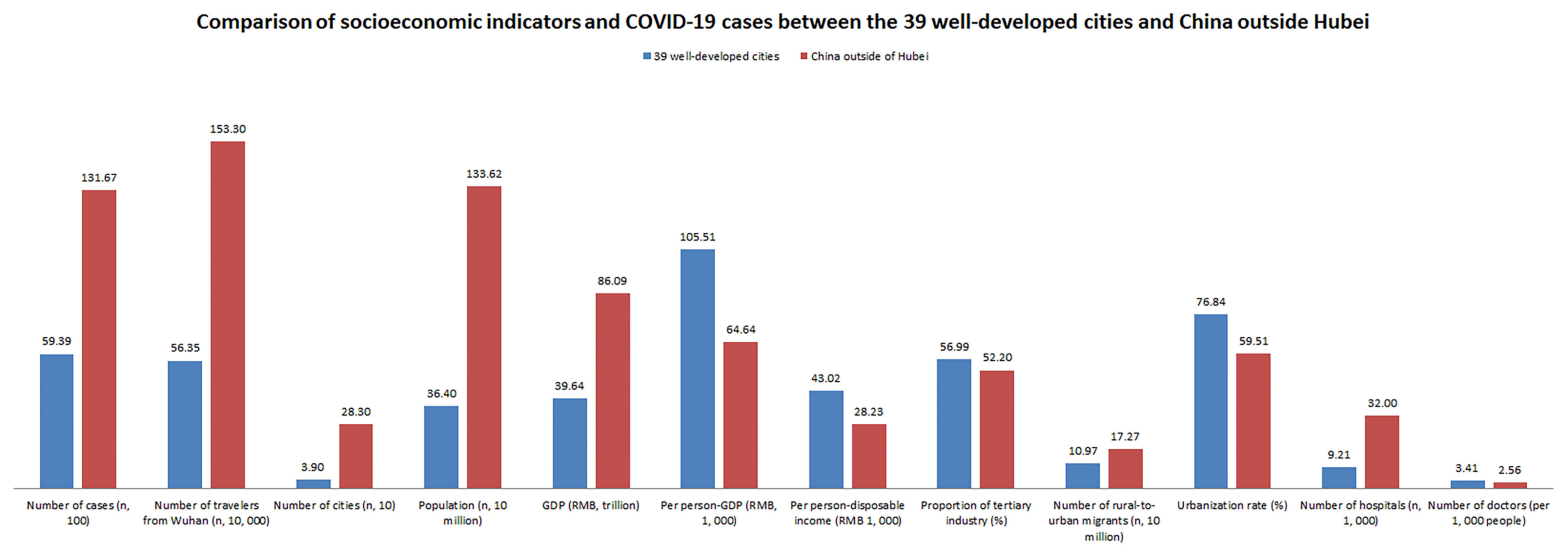
Comparison of socioeconomic indicators and COVID-19 cases between the 39 well-developed cities and China outside Hubei. The 39 well-developed cities only accounted for 13.78% of the national cities except Hubei, but they owned almost a third of the total population and a half of the national GDP or COVID-19 cases.

As of 11:59 P.M. on Mar 19, 2020, a total of 80,967 cases were confirmed infection of COVID-19 in China mainland with 71,150 recovered cases. Of these, a total of 13,167 cases were reported in China outside of Hubei with 5,939 cases (45.11%) in the 39 well-developed cities. Figure 2 shows comparisons of socioeconomic indicators and COVID-19 cases between the 39 well-developed cities and China outside of Hubei. The 39 cities only accounted for 13.78% of the national cities except Hubei, but they owned almost a third of the total population and a half of the national GDP. In addition, there were almost 110 million rural-to-urban migrants (63.54%) in these cities.

### The Correlations Between the Number of Cases and Socioeconomic Factors in the 39 Well-Developed Cities

**Figure 3:**
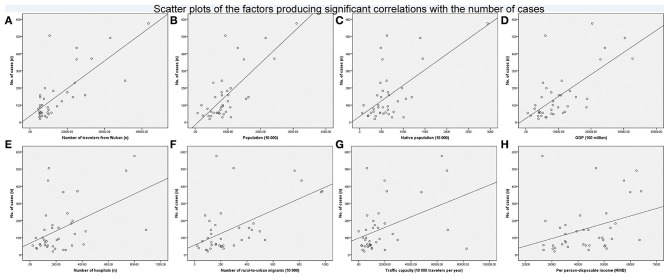
Scatter plots of the factors producing significant correlations with the number of cases. There was a positive correlation between the number of cases and the number of travelers from Wuhan [*r* = 0.663, *P* = 0.000 **(A)**], population [*r* = 0.584, *P* = 0.000 **(B)**], native population [*r* = 0.411, *P* = 0.009 **(C)**], GDP [*r* = 0.596, *P* = 0.000 **(D)**], the number of hospitals [*r* = 0.369, *P* = 0.021 **(E)**], the number of rural-to-urban migrants [*r* = 0.483, *P* = 0.000 **(F)**], traffic capacity [*r* = 0.380, *P* = 0.017 **(G)**], or per-person disposable income [*r* = 0.340, *P* = 0.034 **(H)**].

There were no correlations between the number of cases and population density (*P* = 0.217), per-person GDP (*P* = 0.076), the number of doctors (*P* = 0.401), the proportion of rural-to-urban migrants (*P* = 0.214), urbanization rate (*P* = 0.281), or the proportion of tertiary industry (*P* = 0.794). Figure 3 shows scatter plots of the factors producing significant correlations with the number of cases. There were positive correlations between the number of cases and the number of travelers from Wuhan (*r* = 0.663, *P* = 0.000 Figure 3A), population (*r* = 0.584, *P* = 0.000 Figure 3B), native population (*r* = 0.411, *P* = 0.009 Figure 3C), GDP (*r* = 0.596, *P* = 0.000 Figure 3D), the number of hospitals (*r* = 0.369, *P* = 0.021 Figure 3E), the number of rural-to-urban migrants (*r* = 0.483, *P* = 0.000 Figure 3F), traffic capacity (*r* = 0.380, *P* = 0.017 Figure 3G), and per-person disposable income (*r* = 0.340, *P* = 0.034 Figure 3H).

### The Independent Risk Factors Most Strongly Associated With the Number of Cases Identified by the Multiple Stepwise Linear Regression Modeling

The number of cases was recognized as the dependent variable, and nine socioeconomic variables including the number of travelers from Wuhan, population, native population, GDP, per-person GDP, the number of hospital, the number of rural-to-urban migrants, traffic capacity, and person-disposable income were recognized as independent variables in the multiple stepwise linear regression model. The results showed that the number of travelers from Wuhan (*t* = 6.746, *P* = 0.000) and the number of rural-to-urban migrants (*t* = 3.766, *P* = 0.001) were the independent risk factors most strongly associated with the number of cases in the 39 well-developed cities. Moreover, the result of collinearity diagnostics indicated that the collinearity between independent factors was acceptable in this multiple linear regression model. Additionally, a well-fitted regression equation was as follows: Y = 0.007X_t_ + 0.200X_m_ (adjusted *R*^2^ = 0.833), in which Y was the number of cases in a selected well-developed city, and X_t_, X_m_ were the number of travelers from Wuhan and the number of rural-to-urban migrants in the selected well-developed city (Table 2).

**Table 2:** Statistical test of the constant and independents in the regression equation.

**Dependent variable**	**Adjusted R square**	**Model**	**Unstandardized coefficients**		**Standardized coefficients beta**			**Collinearity statistics**	
			**B**	**Std error**		***t***	***P***	**Tolerance**	**VIF**
Number of cases (*n*=5,939)	0.833	Number of travelers from Wuhan (*n*)	0.007	0.001	0.631	6.746	0.000	0.488	2.049
		Number of rural-to-urban migrants (10, 000)	0.200	0.053	0.353	3.776	0.001	0.488	2.049

## Discussion

Economic growth, urbanization, and globalization pose considerable challenges for the prevention of emerging infectious diseases in China, especially in the well-developed cities (8). In China, the socioeconomic development is highly concentrated, usually in the eastern and southern regions. As we showed, the 39 well-developed cities owned almost a third of the total population and a half of the national GDP. More generally, well-developed cities often function as highways for “microbial traffic” owing to the high level of socioeconomic development (5). Besides, rapid urbanization in cities not only boosts certain well-established infectious diseases (e.g., tuberculosis, dengue) but also facilitates dissemination of various emerging infectious diseases (e.g., the outbreak of SARS in the high-rise housing of Hong Kong) (5, 9). Our results indicated that almost half of the confirmed cases in China outside of Hubei were reported in the 39 well-developed cities, which were only about 13% of the total number of cities in China. Of these, several metropolises such as Beijing, Shanghai, Shenzhen, and Guangzhou reported a large number of confirmed cases with over 350 COVID-19 cases. Therefore, the prevention and control of emerging infectious diseases presents a great pressure on these well-developed cities.

Surprisingly, several classical contributors such as population, population density, income, urbanization rate, and level of health care were not the major risk factors of the spread of COVID-19 in these cities. On the one hand, this result may be explained by the fact that the COVID-19 outbreak just occurred during the Spring Festival holiday in China (10). Since most economic activity was suspended during the Spring Festival holiday in China, the epidemic characteristics of SARS-Cov-2 during the period may not be the same as usual. On the other hand, the cities included in this study were all at a high level of socioeconomic development and there was a relatively small gap in socioeconomic development among them. For instance, per-person GDP and per-person disposable income of these cities were at a high level with a narrow interquartile range in this study. This small gap might underestimate the impact of the above classical factors on the COVID-19 outbreak. Additionally, it was reported that the strict control strategies implemented in Chinese government could reduce social mixing on the outcome of the COVID-19 epidemic (11). Therefore, the impact of these classical contributors on the spread of COVID-19 might be weakened due to the above reasons.

Travelers play a crucial role in importing emerging infectious diseases and could also be a sentinel of major epidemic, and COVID-19 is no exception (12, 13). For the 39 well-developed cities, travelers from Wuhan were also a major risk factor for the COVID-19 outbreak. This result further confirmed our previous finding (2). In agreement with our result, it was suggested that the correlation between the number of COVID-19 cases and the passengers from Wuhan was also positive at a provincial level (14). Therefore, travelers form an epicenter should be paid more attention.

It is interesting to note that rural-to-urban migrants were independently associated with the COVID-19 outbreak in the 39 well-developed cities. Rural-to-urban migrants (also called floating population) emerged in the 1980s when Chinese opening up strategy and economic reforms started, and the number increased every year in China. It was estimated that over 15 million rural-to-urban migrants moved annually from their villages to cities, especially to the well-developed cities in the 21st century (15). In our study, there were 17.27 million rural-to-urban migrants in China in 2018, 63.54% of which were in the 39 well-developed cities. In fact, rural-to-urban migrants usually face substantial restrictions in accessing subsidized housing, social welfare benefits, medical care, and insurance in urban regions, at least partly owing to the household registration (*hukou*) in China (16–18). Rural-to-urban migrants were thought to be more vulnerable to infectious diseases such as tuberculosis, acquired immune deficiency syndrome (AIDS), and sexually transmitted diseases (STDs) (17, 19). Meanwhile, at a nationwide level among the general population, an increase in rural-to-urban migration is associated with the proliferation of infectious diseases in the urban regions of China (20). Recently, the impact of COVID-19 on migrant workers has been received particular attention (21, 22). In line with our results, 88% of nationally confirmed cases occurred among low-skilled migrant workers living in foreign worker dormitories in Singapore, highlighting migrant workers as a vulnerable group for COVID-19 (22). In reality, migrant workers also appear to be at a higher risk for certain infectious diseases at an international level (23). It was suggested that unfavorable working and live conditions, low awareness of disease prevention, accessing inadequate public health services, lower immunization status, and lower socioeconomic status are the possible contributors to the vulnerability of rural-to-urban migrants to acquire infectious diseases in urban regions (24, 25). However, much is unknown about the exact number of rural-to-urban migrants who suffering COVID-19 in these well-developed cities owing to the limited epidemiological data at present. Thus, whether rural-to-urban migrants have higher prevalence of COVID-19 is still unknown. This will be an important issue for future research. Moreover, since internal migration is another model of travel, a large number of rural-to-urban migrants in one city commonly reflect a very high mobility of its population, which has been long associated with spread of emerging infectious diseases (26). In any case, rural-to-urban migrants should be paid more attention in the early stage of the COVID-19 outbreak. Additionally, to reduce the proliferation of infectious diseases, promoting the health and welfare of rural-to urban migrants and their families through more equitable socioeconomic policies and effective public health measures is urgently needed in the well-developed cities (24). Furthermore, a well-fitted regression model was built in this study (adjusted *R*^2^ = 0.833). Based on this model, the number of cases increased by 0.007 with one increase in travelers from Wuhan and increased by 0.200 with 10,000 increases in rural-to-urban migrants in the 39 well-developed cities. Perhaps this regression equation could also serve as a feasible estimated model of COVID-19 cases in a well-developed city where had a similar epicenter and the same characteristics of population mobility as China.

Traditionally, most of rural-to-urban migrants in the well-developed cities would return to their hometown to reunite with their family or visit friends and relatives before the Spring Festival in China. It is puzzling that how rural-to-urban migrants affected the COVID-19 outbreak, as most of them had left these cities during that period. One possible explanation is that not all rural-to-urban migrants returned back to their hometown obviously, and those who were left behind were still a high-risk population for suffering COVID-19. In spite of that, the exact number of rural-to-urban migrants who were left behind in these cities during the Spring Festival holiday is not clear. There are, however, other possible explanations such as the reverse Spring Festival travel rush, which describes a phenomenon where more parents travel from their hometowns to the well-developed cities to visit their children who work there. It was reported that the volume of reverse migration was mainly concentrated in the well-developed cities, especially in major cities such as Beijing, Shanghai, and Guangzhou which owned about 2.5–5.0 million travelers in each during the reverse rush in 2018 (27). A subgroup of travelers from Wuhan which might be potential virus carriers also belonged to this special population with reverse traffic flow. Furthermore, a proportion of confirmed cases were also observed to occur in this special population in Tianjin city according to the official situation report (http://wsjk.tj.gov.cn/).

## Limitations

Despite the intriguing findings of our study, several important limitations should be considered. To begin with, the fact that not all well-developed cities of China were included in this study may underreport some risk factors. Second, our results may be an underestimation as not all cases might be identified and asymptomatic carriers exist. Third, whether rural-to-urban migrants have higher prevalence of COVID-19 is still unknown. Accordingly, the exact reason for the impact of rural-to-urban migrants on the COVID-19 outbreak in the well-developed city is still not clear. Further studies, which take these variables into account, will need to be undertaken. Fourth, due to the effective control of the COVID-19 outbreak in China, the total number of cases included in the study is a bit small, with an average of only about 100 people per city. The small number of cases in each city led to a very low incidence rate, and it may underestimate the impact of the potential contributors on the spread of COVID-19. Lastly but not the least, only several socioeconomic variables were considered in this study, while other potential factors are still not well-explored owing to the limited data at present. All these limitations will lead to an inadequate explanation on the role of socioeconomic factors in the spread of COVID-19 in the well-developed cities. Thus, the results in the present study still need further confirmation.

## Conclusions

In conclusion, travelers from Wuhan and rural-to-urban migrants were independently associated with the COVID-19 outbreak in the 39 well-developed cities of China. Thus, travelers from an epicenter and rural-to-urban migrants should be paid more attention in the early stage of the COVID-19 outbreak in the well-developed cities. These findings could also provide valuable information for other countries to control the future spread of nationwide epidemics, especially for countries which have the same characteristics of population mobility as China.

## Data Availability Statement

All datasets presented in this study are included in the article/supplementary material.

## Author Contributions

PZ conceived, designed the research, and initiated and organized this study. PZ and TC performed the research. Data were analyzed by YL, PZ, and TC. YL and PZ drafted the manuscript. All authors reviewed and edited the manuscript and approved the final version of the manuscript.

## Conflict of Interest

The authors declare that the research was conducted in the absence of any commercial or financial relationships that could be construed as a potential conflict of interest.
